# Supplementary material for: A retrospective prognostic evaluation using unsupervised learning in the treatment of COVID-19 patients with hypertension treated with ACEI/ARB drugs
Source: PeerJ. 2024 May 13;12:e17340. doi: 10.7717/peerj.17340 (PMC11097962; doi:10.7717/peerj.17340)
Supplement: Supplemental Information 1 [file peerj-12-17340-s001.docx]

Table S1 Difference analysis results of the baseline information of the two groups of patients

| variable | Good prognosis (n=47) | Poor prognosis (n=37) | *t*(χ^2^) | *P* |
| --- | --- | --- | --- | --- |
| Age (x±s, years old) | 63.11±13.72 | 74.78±13.03 | -3.959 | <0.001 |
| Gender[n(%)] |  |  | (0.820) | 0.365 |
| male | 25(60.98) | 16(39.02) |  |  |
| female | 22 (51.16) | 21(48.84) |  |  |
| Use ACEI/ARB[n(%)] |  |  | (12.189) | <0.001 |
| yes | 32(74.42) | 11(25.58) |  |  |
| no | 15(36.59) | 26 (63.41) |  |  |
| Hypertension grade [n(%)] |  |  | (6.225) | 0.044 |
| Level 1 | 21(75.00) | 7(25.00) |  |  |
| level 2 | 18 (47.37) | 20(52.63) |  |  |
| Level 3 | 8(44.44) | 10(55.56) |  |  |
| Types of COVID-19[n(%)] |  |  | (3.952) | 0.047 |
| light and normal | 47(58.02) | 34(41.98) |  |  |
| heavy | 0(0) | 3(100) |  |  |
| MuLBSTA (x±s, points) | 5.64±2.87 | 7.27±3.45 | -2.365 | 0.020 |

Note: n (%) represents the number and percentage of cases in the group.

Table S2 Difference analysis results of clinical symptoms between the two groups

| variable | Good prognosis (n=47) | Poor prognosis (n=37) | *x*^2^ | *P* |
| --- | --- | --- | --- | --- |
| Fever [n(%)] |  |  | 0.004 | 0.949 |
| yes | 13 (56.52) | 10(43.48) |  |  |
| no | 34(55.74) | 27 (44.26) |  |  |
| Cough[n(%)] |  |  | 3.736 | 0.053 |
| yes | 36(63.16) | 21(36.84) |  |  |
| no | 11(40.74) | 16 (59.26) |  |  |
| Coughing up sputum[n(%)] |  |  | 0.131 | 0.717 |
| yes | 21(53.85) | 18(46.15) |  |  |
| no | 26(57.78) | 19 (42.22) |  |  |
| Shortness of breath [n(%)] |  |  | 2.788 | 0.095 |
| yes | 1(20.00) | 4(80.00) |  |  |
| no | 46(58.23) | 33 (41.77) |  |  |
| Chest tightness [n(%)] |  |  | 0.075 | 0.784 |
| yes | 6(60.00) | 4(40.00) |  |  |
| no | 41 (55.41) | 33 (44.59) |  |  |
| Fatigue [n(%)] |  |  | 0.001 | 0.980 |
| yes | 5(55.56) | 4 (44.44) |  |  |
| no | 42(56.00) | 33(44.00) |  |  |
| Muscle soreness[n(%)] |  |  | 0.035 | 0.851 |
| yes | 3(60.00) | 2(40.00) |  |  |
| no | 44(55.70) | 35(44.30) |  |  |
| Dizziness [n(%)] |  |  | 0.470 | 0.493 |
| yes | 6 (66.67) | 3 (33.33) |  |  |
| no | 41 (54.67) | 34(45.33) |  |  |
| Sore throat[n(%)] |  |  | 0.473 | 0.491 |
| yes | 9(64.29) | 5(35.71) |  |  |
| no | 38(54.29) | 32(45.71) |  |  |
| Confused [n(%)] |  |  | 0.618 | 0.432 |
| yes | 3(75.00) | 1(25.00) |  |  |
| no | 44(55.00) | 36(45.00) |  |  |
| Anorexia[n(%)] |  |  | 0.742 | 0.389 |
| yes | 5(71.43) | 2 (28.57) |  |  |
| no | 42 (54.55) | 35(45.45) |  |  |
| Diarrhea [n(%)] |  |  | 1.633 | 0.201 |
| yes | 1(25.00) | 3(75.00) |  |  |
| no | 46(57.50) | 34(42.50) |  |  |

Note: n (%) represents the number and percentage of cases in the group.

Table S3 Difference analysis results of laboratory tests between the two groups of patients

| variable | Good prognosis (n=47) | Poor prognosis (n=37) | *z* | *P* |
| --- | --- | --- | --- | --- |
| White blood cells [M(IQR)] | 4.70(1.80) | 5.20(2.55) | -0.158 | 0.875 |
| Neutrophils [M(IQR)] | 2.73 (1.44) | 2.96(1.55) | -0.707 | 0.479 |
| Eosinophils [M(IQR)] | 0.08(0.70) | 0.07(0.60) | -0.660 | 0.510 |
| Lymphocytes [M(IQR)] | 1.39(0.60) | 1.08(0.60) | -2.077 | 0.038 |
| CRP[M(IQR)] | 3.27(5.77) | 8.80(26.56) | -2.822 | 0.005 |
| IL-6[M(IQR)] | 6.40(7.80) | 12.00 (21.20) | -2.748 | 0.006 |
| ESR [M(IQR)] | 14(10) | 18(15) | -1.867 | 0.062 |
| Lactate dehydrogenase [M(IQR)] | 188(43) | 226(77) | -2.636 | 0.008 |
| D-2mer [M(IQR)] | 0.39(0.37) | 0.75(0.66) | -2.586 | 0.010 |
| Oxygen saturation [M(IQR)] | 0.98(0.04) | 0.96(0.03) | -2.599 | 0.009 |
| PCT[M(IQR)] | 0.03(0.03) | 0.07(0.14) | -3.937 | <0.001 |
| AST[M(IQR)] | 21(10) | 25(28) | -2.061 | 0.039 |
| ALT[M(IQR)] | 17(8) | 18(22) | -0.532 | 0.595 |
| Total Bilirubin [M(IQR)] | 9.30(7.10) | 11.40(6.70) | -1.072 | 0.284 |
| Creatinine [M(IQR)] | 87.49 (22.89) | 68.65 (32.27) | -3.059 | 0.002 |
| Ferritin [M(IQR)] | 214.80(187.22) | 198.30(170.09) | -0.185 | 0.853 |
| NT-proBNP[M(IQR)] | 77.83 (254.05) | 138.21 (367.72) | -2.196 | 0.028 |
| Myoglobin [M(IQR)] | 23.48 (14.23) | 29.53 (35.02) | -1.536 | 0.124 |
| High-sensitivity troponin I [M(IQR)] | 0.02(0.00) | 0.02(0.01) | -2.498 | 0.012 |
| N gene [M(IQR)] | 24.17(8.38) | 21.88 (6.68) | -1.951 | 0.051 |
| O gene [M(IQR)] | 22.41 (10.27) | 19.50 (6.69) | -2.194 | 0.028 |

Note: Mann‒Whitney test is used. M represents the median, IQR represents the quartile range.

Table S4 Difference analysis results of imaging examinations between the two groups of patients

| variable | Good prognosis (n=47) | Poor prognosis (n=37) | *x*^2^ | *P* |
| --- | --- | --- | --- | --- |
| Degree of lung involvement [n(%)] |  |  | 5.020 | 0.170 |
| 1 lobe involved | 5(50.00) | 5(50.00) |  |  |
| 2 lobes involved | 33 (64.71) | 18(35.29) |  |  |
| 3 lung lobes involved | 9(40.91) | 13 (59.09) |  |  |
| 4 lung lobes involved | 0(0.00) | 1(100.00) |  |  |
| Pleural effusion[n(%)] |  |  | 0.789 | 0.374 |
| yes | 10(47.62) | 11(52.38) |  |  |
| no | 37 (58.73) | 26 (41.27) |  |  |
| Patchy and cord-like increased density [n(%)] |  |  | 2.836 | 0.092 |
| yes | 18(46.15) | 21(53.85) |  |  |
| no | 29 (64.44) | 16(35.56) |  |  |
| Ground glass nodules [n(%)] |  |  | 0.909 | 0.340 |
| yes | 7(70.00) | 3(30.00) |  |  |
| no | 40(54.05) | 34(45.95) |  |  |
| Consolidation[n(%)] |  |  | 0.195 | 0.659 |
| yes | 8(61.54) | 5(38.46) |  |  |
| no | 39 (54.93) | 32(45.07) |  |  |

Note: n (%) represents the number and percentage of cases in the group. The imaging examination in this section is X-ray.
