# Supplementary material for: A retrospective prognostic evaluation using unsupervised learning in the treatment of COVID-19 patients with hypertension treated with ACEI/ARB drugs
Source: PeerJ. 2024 May 13;12:e17340. doi: 10.7717/peerj.17340 (PMC11097962; doi:10.7717/peerj.17340)
Supplement: Supplemental Information 2 [file peerj-12-17340-s002.docx]

**System demonstration**

This software is developed based on MATLAB R2022a and utilizes the APP Designer function to design the original *. mlapp file. On this basis, MATLAB's built-in MATLAB Runtime compiler is used to compile the *. mlapp file into a *. exe file that can be executed independently of the MATLAB environment. As long as the software is pre installed on a computer with MATLAB Runtime, it can be run, successfully reducing the running environment requirements of the software and improving portability.

(1) Hardware requirements

Processor main frequency: 2Ghz and above;

Memory: 2GB or above.

(2) Software requirements

System: Windows 10 and above (64 bit system);Operating environment: MATLAB R2022a and above

MATLAB Runtime version 9.12 and above.

First, double-click on the running software to enter the system interface.

(1) After successfully initializing and configuring the user environment, the system login interface of the software will be displayed (Figure 1). The user enters the correct account and password, clicks the "login button", and enters the initial system operation interface (Figure 2)

(2) The user inputs the data corresponding to the six characteristic variables of the patient, and then clicks the "Start Prediction" button. The system will automatically calculate the prediction result based on the characteristic variables and output it in the text box, as shown in Figure 3.

(3) Click the "Result Storage" button to save the data (Figure 4), click the "Cleaning" button to clean the interface, return to the initial interface, and predict the results of new patients.


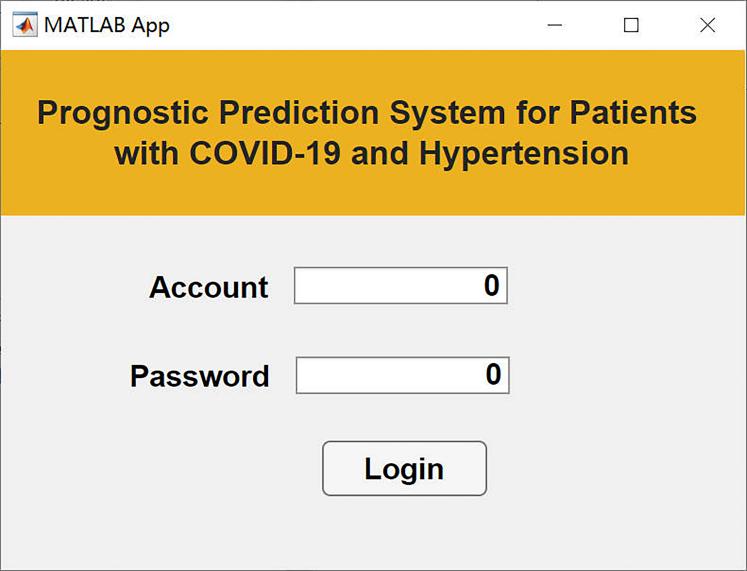


Figure 1 System Login Interface


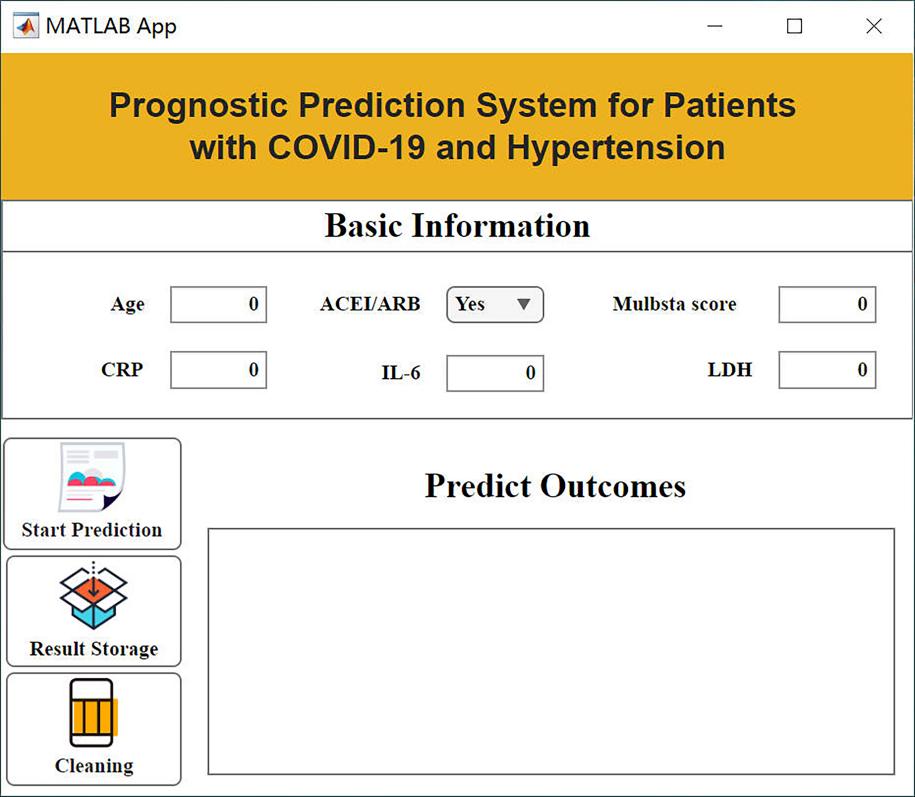


Figure 2 System Initial Operation Interface


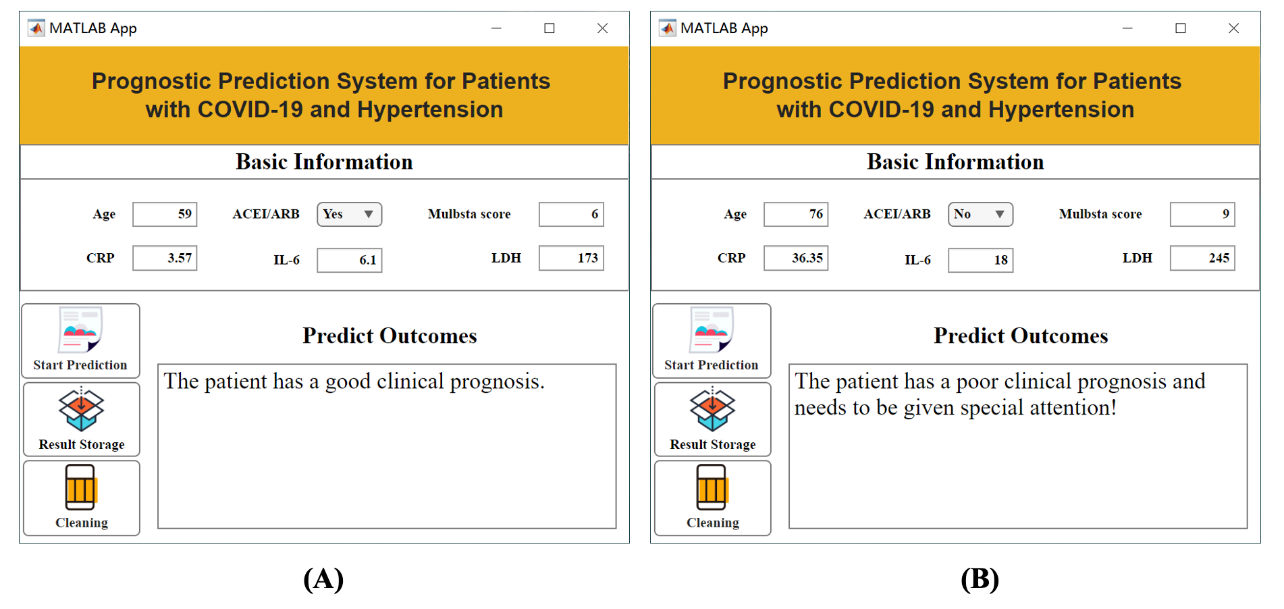


Figure 3 Display of Prediction Results

Note: (A) Examples of patients with good prognosis; (B) Examples of patients with poor prognosis.


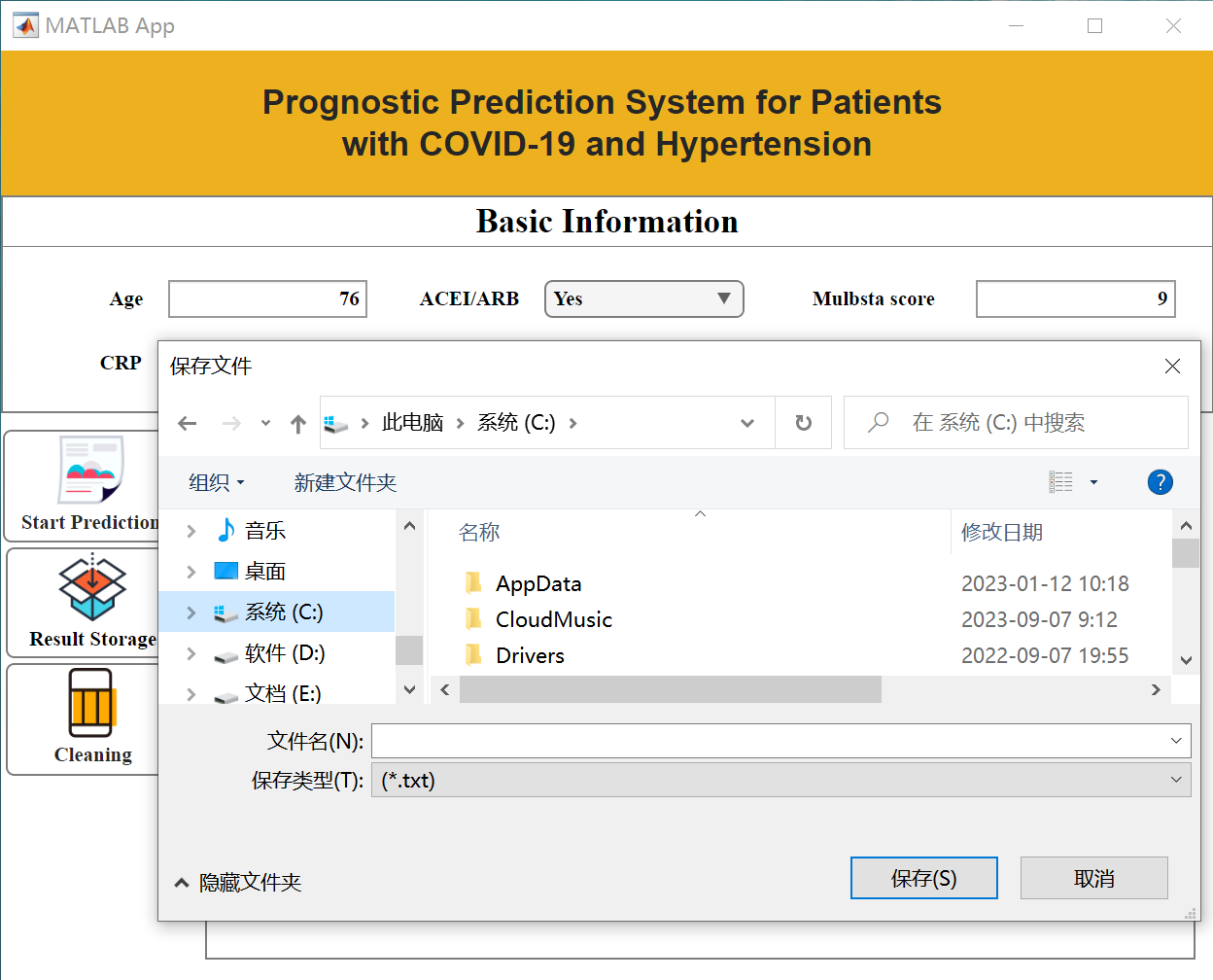


Figure 4 Data Saving Demonstration
