# Supplementary material for: A retrospective prognostic evaluation using unsupervised learning in the treatment of COVID-19 patients with hypertension treated with ACEI/ARB drugs
Source: PeerJ. 2024 May 13;12:e17340. doi: 10.7717/peerj.17340 (PMC11097962; doi:10.7717/peerj.17340)
Supplement: Supplemental Information 3 [file peerj-12-17340-s003.docx]

**Standard function**

**Characteristics of 23 standard test functions**

| **Number** | **Name** | **The boundary range of the search space** | **Function dimension** | **Functional category** | **Optimal solution** |
| --- | --- | --- | --- | --- | --- |
| F1 | Sphere | [-100,100] | 30 | U | 0 |
| F2 | Schwefel’s problem 2.22 | [-100,10] | 30 | U | 0 |
| F3 | Schwefel’s problem 1.2 | [-100,100] | 30 | U | 0 |
| F4 | Schwefel’s problem 2.21 | [-100,100] | 30 | U | 0 |
| F5 | Rosenbrock | [-30,30] | 30 | U | 0 |
| F6 | Step | [-100,100] | 30 | U | 0 |
| F7 | Noise | [-128,128] | 30 | M | 0 |
| F8 | Generalized Schwefel’s problem | [-500,500] | 30 | M | -12569.5 |
| F9 | Rastrigin | [-5.12,5.12] | 30 | M | 0 |
| F10 | Ackley | [-32,32] | 30 | M | 0 |
| F11 | Griewan | [-600,600] | 30 | M | 0 |
| F12 | Generalized Penalized Function 1 | [-50,50] | 30 | M | 0 |
| F13 | Generalized Penalized Function 2 | [-50,50] | 30 | M | 0 |
| F14 | Shekel’s Foxholes function | [-65,65] | 2 | M | 1 |
| F15 | Kowalik’s function | [-5,5] | 4 | M | 0.0003 |
| F16 | Six-hump camel back | [-5,5] | 2 | M | -1.0316 |
| F17 | Branin | [-5,5] | 2 | M | 0.398 |
| F18 | Goldstein-Price function | [-2,2] | 2 | M | 3 |
| F19 | Hartman 1 | [1,3] | 3 | M | -3.86 |
| F20 | Hartman 2 | [0,1] | 6 | M | -3.32 |
| F21 | Shekel 1 | [0,10] | 4 | M | -10.1532 |
| F22 | Shekel 2 | [0,10] | 4 | M | -10.4028 |
| F23 | Shekel 3 | [0,10] | 4 | M | -10.5363 |

**Formulas for 23 standard test functions**

| **Number** | **Functional expression** |
| --- | --- |
| 1 | $\sum_{i=1}^{n} x_{i}^{2}$ |
| 2 | $\sum_{i=1}^{n} \left\vert x_{i}\right\vert+\prod_{i=1}^{n} \left\vert x_{i}\right\vert$ |
| 3 | $\sum_{i=1}^{n} \left(\sum_{j-1}^{i} x_{j}\right)^{2}$ |
| 4 | $\max_{i}\left\{\left\vert x_{i}\right\vert,1\leq i\leq n\right\}$ |
| 5 | $\sum_{i=1}^{n-1} \left[100\left(x_{i+1}-x_{i}^{2}\right)+\left(x_{i}-1\right)^{2}\right]$ |
| 6 | $\sum_{i=1}^{n} \left(\left[x_{i}+0.5\right]\right)^{2}$ |
| 7 | $\sum_{i=1}^{n} ix_{i}^{4}+random\left[0,1\right]$ |
| 8 | $\sum_{i=1}^{n} -x_{i}\sin\left(\sqrt{\left\vert x{}_{i}\right\vert}\right)$ |
| 9 | $\sum_{i=1}^{n} \left[x_{i}^{2}-10\cos\left(2\pi x_{i}\right)+10\right]$ |
| 10 | $-20\exp\left(-0.2\sqrt{\frac{1}{n}\sum_{i=1}^{n} x_{i}^{2}}\right)-\exp\left(\frac{1}{n}\sum_{i=1}^{n} \cos\left(2\pi x_{i}\right)\right)+20+e$ |
| 11 | $\frac{1}{4000}\sum_{i=1}^{n} x_{i}^{2}-\prod_{i+1}^{n} \cos\left(\frac{x_{i}}{\sqrt{i}}\right)+1$ |
| 12 | $\frac{\pi}{n}\left\{10\sin\left(\pi y_{1}\right)+\sum_{i=1}^{n-1} \left(y_{i}-1\right)^{2}\left[1+10\sin^{2}\left(\pi y_{i+1}\right)\right]+\left(y_{n}-1\right)^{2}\right\}+\sum_{i=1}^{n} u\left(x_{i},10,100,4\right)$ $y_{i}=1+\frac{x_{i}+1}{4}$   , $u(x_{i},a,k,m)=\left\{\begin{matrix} k\left(x_{i}-a\right)^{m}\text{ }x_{i}>a \\ 0-a\text{ }0<x_{i}<a \\ k\left(-x_{i}-a\right)^{m}\text{ }x_{i}>a \end{matrix}\right.$ |
| 13 | $0.1\left\{\sin^{2}\left(3\pi x_{1}\right)+\sum_{i=1}^{n} \left(x_{i}-1\right)^{2}\left[1+\sin^{2}\left(3\pi x_{i}+1\right)\right]+\left(x_{n}-1\right)^{2}\left[1+\sin^{2}\left(2\pi x_{n}\right)\right]\right\}+\sum_{i=1}^{n} u\left(x_{i},5,100,4\right)$ |
| 14 | $\left(\frac{1}{500}+\sum_{j=1}^{25} \frac{1}{j+\sum_{i=1}^{2} \left(x_{i}-a_{ij}\right)^{6}}\right)^{-1}$ |
| 15 | $\sum_{i=1}^{11} \left[a_{i}-\frac{x_{1}\left(b_{i}^{2}+b_{i}x_{2}\right)}{b_{i}^{2}+b_{i}x_{3}+x_{4}}\right]^{2}$ |
| 16 | $4x_{_{1}}^{2}-2.1x_{_{1}}^{4}+\frac{1}{3}x_{_{1}}^{6}+x_{1}x_{2}-4x_{_{2}}^{2}+4x_{2}^{4}$ |
| 17 | $\left(x_{2}-\frac{5.1}{4\pi^{2}}x_{1}^{2}+\frac{5}{\pi}x_{1}-6\right)^{2}+10\left(1-\frac{1}{8\pi}\right)\cos x_{1}+10$ |
| 18 | $\left[1+\left(x_{1}+x_{2}+1\right)^{2}\left(19-14x_{1}+3x_{1}^{2}-14x_{2}+6x_{1}x_{2}+3x_{2}^{2}\right)\right]\times$ $\left[30+\left(2x_{1}-3x_{2}\right)^{2}\times\left(18-32x_{1}+12x_{_{1}}^{2}+48x_{2}-36x_{1}x_{2}+27x_{2}^{2}\right)\right]$   |
| 19 | $-\sum_{i=1}^{4} c_{i}\exp\left(-\sum_{j=1}^{3} a_{ij}\left(x_{j}-p_{ij}\right)^{2}\right)$ |
| 20 | $-\sum_{i=1}^{4} c_{i}\exp\left(-\sum_{j=1}^{6} a_{ij}\left(x_{j}-p_{ij}\right)^{2}\right)$ |
| 21 | $-\sum_{i=1}^{5} \left[\left(X-a_{i}\right)\left(X-a_{i}\right)^{T}+c_{i}\right]^{-1}$ |
| 22 | $-\sum_{i=1}^{7} \left[\left(X-a_{i}\right)\left(X-a_{i}\right)^{T}+c_{i}\right]^{-1}$ |
| 23 | $-\sum_{i=1}^{10} \left[\left(X-a_{i}\right)\left(X-a_{i}\right)^{T}+c_{i}\right]^{-1}$ |
